# Integrating Mobile Health App Data Into Electronic Medical or Health Record Systems and Its Impact on Health Care Delivery and Patient Health Outcomes: Scoping Review

**DOI:** 10.2196/66650

**Published:** 2025-06-23

**Authors:** Jialing Lin, Shona Marie Bates, Luke N Allen, Michael Wright, Limin Mao, Michael Kidd

**Affiliations:** 1 International Centre for Future Health Systems, University of New South Wales, Level 2, AGSM Building, UNSW Sydney, Kensington, 2052, Australia, 61 02 9348 1394, 61 02 9348 1394; 2 School of Population Health, Faculty of Medicine and Health, University of New South Wales (Sydney), Kensington, Australia; 3 Nuffield Department of Primary Care Health Sciences, University of Oxford, Oxford, United Kingdom; 4 Avant Mutual, Sydney, Australia; 5 Centre for Health Economics Research and Evaluation, University of Technology Sydney, Sydney, Australia; 6 Centre for Social Research in Health, University of New South Wales, Kensington, Australia; 7 College of Health & Medicine, Australian National University, Canberra, Australia; 8 Department of Family and Community Medicine, University of Toronto, Toronto, ON, Canada

**Keywords:** mHealth, digital health, mobile phones, electronic medical records, integration, usability, implementation, scoping review

## Abstract

**Background:**

Mobile health (mHealth) apps are increasingly being used to capture patient health data, provide information, and guide self-management, with reported improvements in health care service delivery and outcomes. However, the impact of integrating mHealth app data into electronic medical record or electronic health record (EMR/EHR) systems remains underexplored.

**Objective:**

This study aims to identify what is known about the impact of integrating mHealth app data into EMR/EHR systems on health care delivery and patient outcomes.

**Methods:**

A scoping review was conducted to identify original studies that investigated the integration of patient-facing mHealth app data into EMR/EHR systems and the impact on health care outcomes. The PubMed, Embase, Web of Science, Cochrane Library, CINAHL, ProQuest, and PsycINFO databases were searched for papers published between January 2014 and July 2024. Two authors independently screened and extracted data on study characteristics, mHealth app features, details of integration with EMR/EHR systems, and effects on health care delivery and patient outcomes.

**Results:**

Nineteen studies with 113,135 participants were included. Among these, 6 were randomized clinical trial studies, 8 were conducted in the United States, 12 occurred in hospital settings, 15 involved adult participants, and 6 targeted diabetes management. Main features of the apps and EMR/EHR systems can be categorized into tracking or recording health data (n=19), app data integrated into EMR/EHR systems (n=19), app data summarized or presented on EMR/EHR interface (n=19), communication with the health care team (n=12), reminders or alerts (n=10), synchronization with other apps or devices (n=8), educational information (n=4), and using existing portal credentials to app access (n=2). Most studies reported benefits of integrating the app and EMR/EHR, such as enhanced patient education and self-management (n=5), real-time data recorded and shared with clinicians (n=4), support for clinical decision-making (n=3), improved communication between patients and clinicians (n=7), and improved patient outcomes (n=13). Challenges identified included high drop-off rates in app usage (n=3), limited accessibility due to device restrictions (n=3), incompatibility between mHealth apps and EMR/EHR systems (n=3), increased clinical workload in response to additional information (n=3), data accuracy issues due to network connectivity (n=1), and data security concerns (n=1).

**Conclusions:**

Evidence suggests that the effective integration of mHealth app data into EMR/EHR systems can enhance both clinicians’ health care delivery and patients’ health outcomes. However, current literature is limited, and future opportunities remain to examine the impact on long-term outcomes, such as mortality, readmissions, and costs, and assess the scalability and sustainability of integration among more broader health conditions and disabilities across diverse health care settings.

## Introduction

### Background

Mobile health (mHealth) apps, defined as mobile devices used to deliver health care, are increasingly being used by patients to manage chronic disease, monitor and record vital signs, self-diagnose, take medication, track general health and wellness, access electronic patient portals, and access educational resources and self-management tools [1,2]. These apps offer personalized health data tracking and can provide valuable insights for both patients and health care providers. For example, some apps designed for chronic disease management, such as those for diabetes, hypertension, cardiovascular diseases, asthma, and other chronic conditions, have had positive impacts on lifestyle modifications, including weight reduction through healthier eating and regular exercise [3]. Additionally, apps focused on monitoring specific aspects of health, such as child nutrition, mental health, and suicide crises, have shown improved health outcomes, including growth in undernourished children, weight loss in overweight or obese individuals, reduced mental health symptoms, and the prevention of suicide [4-6].

Over the past decade, significant progress has been made in developing interoperability frameworks that have the capacity to connect mHealth solutions with electronic medical record (EMR, defined as a digital version of a patient’s medical chart within a single health care facility) or electronic health record (EHR, defined as a comprehensive digital record of a patient’s health that can be shared across multiple health care providers and facilities) systems [7-9]. While these technical advancements are essential, integration in health care goes beyond interoperability. It also includes clinical (eg, shared care planning and coordination), functional (eg, harmonized workflows and service delivery processes), and organizational dimensions (eg, collaboration across different service providers and governance structures) [10]. Incorporating data from patients’ mHealth apps into EMR/EHR systems may enhance the continuity of care by capturing health information between patient visits [11]. This broader integration can provide a more comprehensive view of a patient’s health, streamline documentation processes of patient history, and reduce the burden of manual data entry for health care providers, thus reducing the time spent on documentation during office visits [12,13]. Furthermore, real-time data access allows for better-informed decision-making and more comprehensive patient care [14,15].

Research on the impact of mHealth-EMR/EHR integration on patient outcomes has yielded mixed results. For example, 1 trial recruited 269 people with diabetes and allocated them into 3 groups: a usual care group that did not use an mHealth app, a mobile self-care group that used an mHealth app allowing users to enter their blood glucose data and receive educational information, and a mobile intensive care group that used an app integrated into EMR/EHR systems, enabling physicians to provide personalized feedback. Both the mobile intensive care and mobile self-care groups experienced significant reductions in hemoglobin A_1c_ levels at 12 weeks compared with the usual care group (−1.04% vs −0.86% vs −0.49%; *P*=.02) [12]. Similarly, a trial involving 68 people with overweight or obesity reported significant weight loss (mean difference 1.4 kg, 95% CI 0.9‐1.9; *P*<.001) and a decline in triglyceride levels (mean difference 2.6 mmol/L, 95% CI 17.6‐75.8; *P*=.002) among app users compared with nonusers [16]. This app automatically collected physical activity data from a wearable tracker and allowed users to log additional health data, such as daily meals, sleep, weight, and blood pressure, which were summarized and displayed as graphs on the EMR/EHR interface.

However, there is incredible diversity in app function, design, and purpose, and some studies have reported no significant clinical outcomes. For example, a cross-sectional study found no significant difference in the number of visits to rheumatologists, or the time between appointments, among people with rheumatoid arthritis who used an app to receive reminders to record their pain, fatigue, and disease symptoms, compared with a control group (*P*>.05) [17]. Additionally, a pilot study involving people with diabetes reported no significant change in hemoglobin A_1c _values between app users, with their glucometer data directly transferred from the app to an EMR/EHR system, and nonusers (*P*=.08) [18].

The inconsistent findings across studies underscore the need for a review of existing evidence on the integration of mHealth and EMR/EHR and health outcomes, in order to obtain insights and develop practical recommendations for health care policy makers, administrators, and providers, guiding the effective implementation of mHealth app data integration with EMR/EHR systems.

### Objectives

This scoping review aimed to synthesize current evidence on how patient-facing mHealth app data can be integrated into EMR/EHR systems and affect health care delivery and patient health outcomes. The hypothesis was that effectively linking mHealth apps with EMR/EHR systems could improve outcomes.

## Methods

This scoping review adhered to the PRISMA-ScR (Preferred Reporting Items for Systematic Reviews and Meta-Analyses Extension for Scoping Reviews) statement [19].

### Study Identification

The scoping review identified original studies that assessed the integration of mHealth app data into EMR/EHR systems and impact on health care delivery and outcomes from January 1, 2014, to July 30, 2024, from the following databases: PubMed, Embase, Web of Science, Cochrane Library, CINAHL, ProQuest, and PsycINFO. Search terms including keywords and subject headings were related to mHealth app (eg, “mobile app,” “mHealth app,” “phone app,” “digital app,” “eHealth app,” and “health app”) and EMR/EHR (eg, “electronic health record,” “electronic medical record,” “electronic health information,” and “electronic medical information”) (see search string details in Multimedia Appendix 1). Additionally, the bibliographies of all included studies were manually checked for additional relevant studies. The retrieved studies were then uploaded into Covidence (Veritas Health Innovation), a systematic review management software program.

### Study Selection and Data Extraction

Two authors (JL and SB) independently reviewed the studies by screening titles or abstracts and screening full texts, and they performed data extraction. Disagreements were resolved by consensus. Studies were included if they met the following criteria: the apps were patient-facing, patient-related health data were recorded in the apps, app data were integrated into EMR/EHR systems, effects on health care delivery and patient health outcomes were assessed, and the studies were peer-reviewed original studies and published in English. Specifically, this review included both third-party apps (developed outside the EMR/EHR ecosystem) and native EMR/EHR-integrated apps (eg, Epic’s offerings). Native EMR/EHR-integrated apps were included because they interact with the EMR/EHR system and serve as comprehensive patient management tools, extending beyond standard EMR/EHR functionality. In addition, studies were excluded if (1) the study design was ineligible: reviews, summaries, research protocols, opinion papers, conference abstracts, correspondence, commentaries, or editorials; (2) the intervention was ineligible: the mHealth app was not patient-faced, did not record health data (eg, appointments or reminders only), consisted of mixed or multiple interventions where the effect of mHealth app use cannot be extracted or identified, or where there was only a mention of integration of app data with EMR/EHR systems; or (3) the outcomes were ineligible: where there was no mention of any health outcomes related to the impact of mHealth app data and their integration with EMR/EHR systems.

Original publications were obtained for papers considered in scope. The following data were extracted into an Excel spreadsheet (version 2408): author, year of publication, study country, study setting, study design, sample size, participant characteristics (eg, age, health condition, etc), app features (eg, app type, health data recording, education, reminder, communication, etc), details of app data integrated into EMR/EHR systems (eg, clinical workflow and efficiency, data interoperability and communication, data quality, system integration, adoption, change management, etc), and effects on health care delivery and patient health outcomes.

### Data Synthesis and Analysis

The results were synthesized and presented descriptively. Key features of mHealth apps and EMR/EHR systems were summarized and visualized as a bar chart using R version 4.4.0 (R Core Team 2017, Vienna, Austria).

## Results

### Study Selection

**Figure 1.**
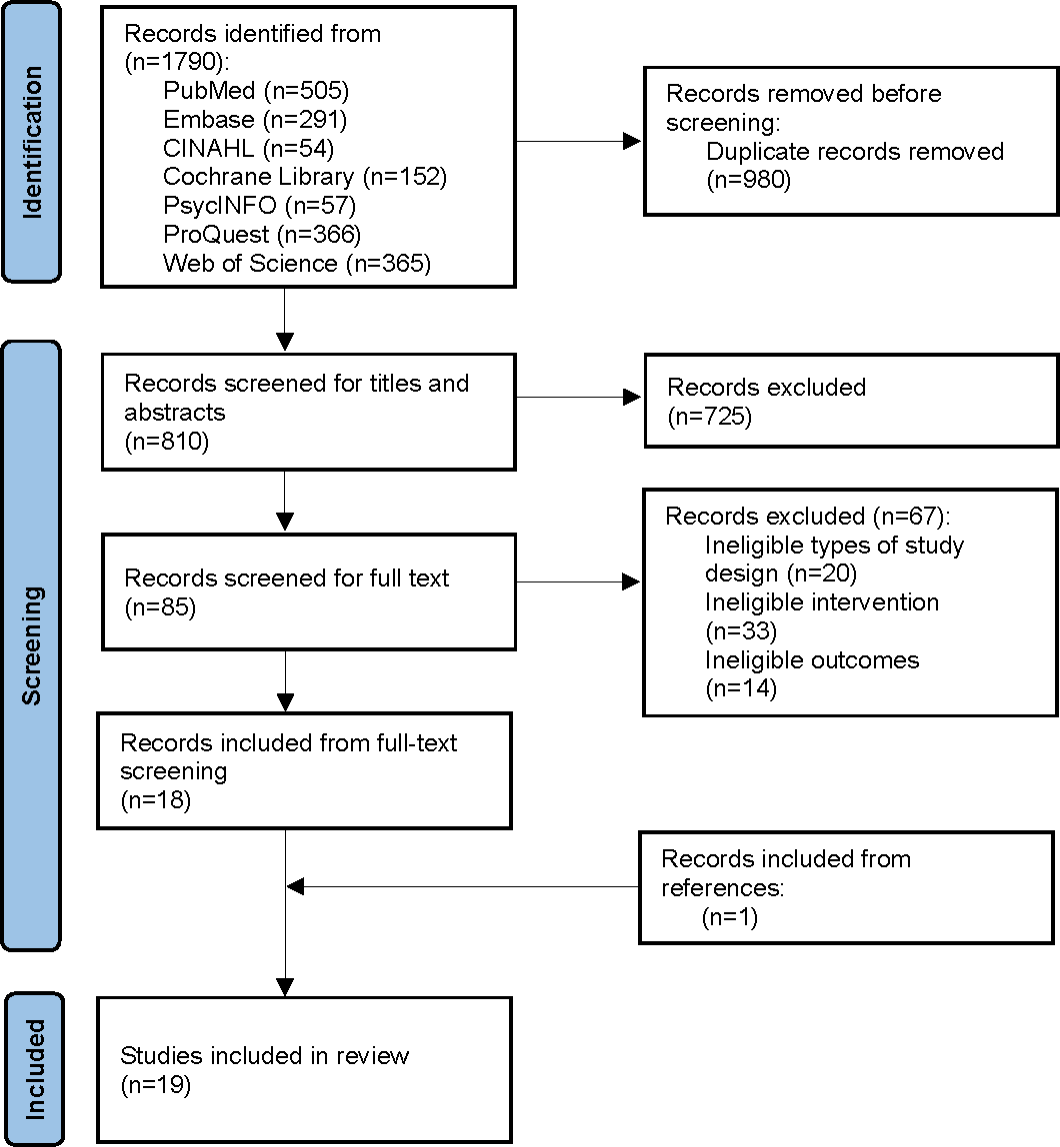
Flow diagram of study selection.

A total of 1790 studies were identified from 7 databases (Figure 1). After removing duplicates, 810 were screened based on and titles and abstracts, resulting in the exclusion of 725 studies that did not meet the inclusion criteria. The remaining 85 studies underwent a full-text review, and 18 studies were deemed eligible. One additional study was included from the bibliography search of the in-scope studies, leaving a total of 19 studies to be included in this review [12-18,20-31].

### Study Characteristics

The 19 studies involved 113,135 participants from 5 countries. Of these, 6 were randomized clinical trials, 6 were cohort studies, 2 were cross-sectional studies, 2 were mixed methods studies, 1 was quasi-experimental study, 1 was case series study, and 1 was qualitative study (Table 1). Approximately two-thirds of studies were based out of hospital settings (n=12). Most studies focused on the adult population (n=15). The 3 most commonly targeted health conditions were diabetes (n=6), cancer (n=3), and obstructive sleep apnea (n=2). Details of the study characteristics are shown in Multimedia Appendix 2.

**Table 1.** Summary of study characteristics.

Study characteristics	Studies, n (references)
Study design	
Randomized control trial	6 [12,14,16,27-29]
Cohort	6 [13,20,23,25,26,31]
Quasi-experimental	1 [15]
Case series	1 [24]
Cross-sectional	2 [17,21]
Qualitative study	1 [30]
Mixed methods	2 [18,22]
Study location	
United States	8 [13,17,18,22-25,30]
United Kingdom	1 [27]
Italy	1 [20]
South Korea	7 [12,14,16,26,28,29,31]
China	2 [15,21]
Study setting	
Hospital	12 [12-15,20,21,23,26-29,31]
Community	7 [16-18,22,24,25,30]
Study population age type	
Adult	15 [12,14-17,20-25,27-29,31]
Children	4 [13,18,26,30]
Study population health diagnosis	
Diabetes	6 [12,13,18,21,30,31]
Hypertension	1 [25]
Heart failure	1 [15]
Cancer	3 [20,24,28]
Overweight/obesity	1 [16]
Rheumatoid arthritis	1 [17]
Chronic insomnia disorder	1 [23]
Obstructive sleep apnea	2 [14,29]
Severe mental illness	1 [27]
Migraine	1 [22]
Epilepsy	1 [26]
Total	19

### Features of mHealth Apps and Their Integrated EMR/EHR Systems

There were 17 unique apps reported in the 19 studies. One app was used in 2 studies [16,29], and another app was used in the other 2 studies [13,30]. The main features of the apps and EMR/EHR systems can be categorized as tracking or recording health data (n=19), app data integrated into EMR/EHR systems (n=19), app data being summarized or presented on EMR/EHR interface (n=19), communicating with the health care team (n=12), providing reminders or alerts (n=10), synchronizing with other apps or devices such as “wearables” (n=8), offering educational resources (n=4), and using existing portal credentials to app access (n=2) (Multimedia Appendix 3). All 17 apps included at least one of these features. Most of the apps were third-party apps developed outside the EMR/EHR ecosystem (n=17), while 2 were native EMR/EHR-integrated apps (ie, Epic’s modules) (n=2) [22,24]. Details of the characteristics of the apps and systems can be found in MultimediaAppendices 2, 4,  5.

### Study Outcomes

Patient outcomes that were explored in the included studies related to blood glucose (n=4) [12,18,21,31], blood pressure (n=2) [15,25], blood triglyceride (n=1) [16], pain intensity (n=2) [20,28], sleep patterns (n=3) [14,23,29], body weight (n=4) [14-16,29], diet (n=1) [15], respiratory distress (n=1) [29], oxygen desaturation (n=1) [29], reactions to treatment (n=1) [28], medication adherence rates (n=2) [15,26], doctor visits (n=1) [17], patient satisfaction (n=2) [15,22], patient health awareness (n=2) [13,15], patient health knowledge (n=1) [26], and patient app usage (n=3) [24,27,28]. Other outcomes reported related to health care providers, such as provider knowledge (n=1) [30], provider access to patient records (n=3) [27,29,30], communication with patients (n=3) [15,25,30], efficiency of patient data interpretation (n=5) [13,25,27,29,30], clinical decision-making (n=5) [15,22,25,27,29], and provider workflow (n=6) [13,15,17,22,25,31].

### Benefits of mHealth App Data Integrated Into EMR/EHR Systems on Outcomes

Most studies reported beneficial effects of mHealth app data integration into EMR/EHR systems (n=13) [12-16,20-23,25-27,29,30]. Specifically, 5 studies reported that the integration enhanced patient education and self-management [12,14,16,21,29]. These studies showed benefits in apps targeting diabetes management [12,21], obstructive sleep apnea [14,29], and weight loss [16] through behavioral interventions and lifestyle changes.

Four studies showed benefits of real-time data recording and sharing with clinicians [13,15,27,30]. Apps with features that enable patients to record or upload health data were found to enhance and sustain patient engagement in their health care. Additionally, 3 studies reported benefits in supporting clinical decision-making [13,15,30], where mHealth apps were directly integrated with devices such as glucometers, blood pressure monitors, and wearable trackers. This enabled automatic data transmission to the app and EMR/EHR systems, allowing health care teams to promptly assess patient health status and make informed therapeutic decisions.

Seven studies reported improved communication between patients and clinicians [12,13,15,20,25,27,30], facilitated by built-in messaging features. Enhanced communication was associated with better-coordinated care, more timely interventions, and improved patient outcomes. Furthermore, EMR/EHR-integrated app-based systems with built-in reminders or alerts were found to support clinicians in managing patient care, enabling timely detection and intervention for changes in patient health status, ultimately leading to better health outcomes [17,21,26,27]. Overall, 13 studies reported better patient health outcomes [12-16,20-23,25-27,29], including reductions in blood glucose levels, blood pressure measurements, chemotherapy-related adverse events, body weights, and seizure frequency.

### Challenges of mHealth App Data Integrated Into EMR/EHR Systems on Outcomes

However, some challenges were identified, including high drop-off rates in app usage due to non–user-friendly interfaces or device breakdowns (n=3) [23,24,26], limited accessibility due to device restrictions (n=3) [16,27,30], data accuracy issues related to network connectivity (n=1) [27], data security concerns (n=1) [18], compatibility problems between mHealth apps and EMR/EHR systems (n=2) [13,27], and increased clinical workload due to receiving additional data (n=3) [15,17,31].

High drop-off rates in app usage were attributed to difficulties in using the apps, the requirement for manual data entry, and device malfunctions, all of which affected compliance and reduced user satisfaction [23,24,26]. Additionally, patient age was reported as a barrier to adopting digital health care and may impact their willingness to use apps in 1 clinical trial conducted in South Korea [12]; this reinforces the importance of consumer involvement to understand their needs and preferences.

Data accuracy remains a concern when it relies heavily on patient self-reported data. A cohort study, involving 99 people with epilepsy and caregivers in a South Korea hospital, found that the accuracy of using apps to measure medication adherence varied significantly depending on patient behavior and engagement with the app when it required users to manually enter data [26].

Data confidentiality concerns also pose a barrier to app adoption and the completeness of data captured. A mixed methods study conducted in a US diabetes clinic, which found no difference in blood glucose levels between people with diabetes who used the app and those who did not, reported that some patients and caregivers expressed concerns about the confidentiality of their data recorded in the app [18]. Ensuring that patient data shared through mHealth apps and EMR/EHR systems remain confidential and adhere to relevant privacy regulations is pivotal.

Broader considerations are needed regarding data integration across different systems. For example, a clinical trial conducted in UK mental health clinics found that app data uploads, which require a wireless network, can be disrupted if users are temporarily in areas without network coverage, leading to mistimed data recording [27].

Compatibility issues between apps and different EMR/EHR platforms present another significant challenge. Two studies found that the use of multiple nonstandardized EMR/EHR platforms within a single health system acted as a barrier to the widespread rollout of integrated solutions across entire health care systems [13,27]. This lack of standardization made it difficult to streamline integration between a single app and different EMR/EHR platforms. A cohort study conducted in a US medical center found that integrating app data into EMR/EHR systems that rely on specific devices, such as Apple Health, requires the use of Apple devices and can limit patient accessibility and usage [13]. Additionally, some apps were available only for 1 operating system, such as Android or iOS (Apple), restricting access for users with mobile devices running different operating systems [16,27,30].

Increased clinical workload and insufficient training for health care providers also emerged as barriers. A cross-sectional study of patients with rheumatoid arthritis in a large US medical center compared 150 app users whose data were integrated into the EMR/EHR system with 150 nonusers [17]. The study reported insufficient training on using EMR/EHR systems with integrated app data, while clinicians’ fully booked schedules hindered their ability to incorporate patient-generated data into clinical workflows and make informed decisions. This may explain the lack of significant differences in health outcomes between app users and nonusers [17]. Furthermore, 2 studies found that an inadequate clinician workforce can hinder the timely provision of feedback by the health care team [15,31], further restricting the potential benefits of mHealth app-EMR integration. Additional details are reported in Multimedia Appendix 6.

## Discussion

### Principal Results

This review presents the first comprehensive synthesis of the literature examining integrating mHealth app data into EMR/EHR systems and its impact on health care outcomes. The 19 studies reported 17 unique patient-facing mHealth apps from 5 countries. The primary features of these apps and EMR/EHR systems include tracking or recording patient health data, integrating app data into EMR/EHR systems, facilitating communication with health care teams, providing reminders or alerts, synchronizing with other apps or devices, displaying summarized app data on EMR/EHR interfaces, using existing portal credentials to app access, and offering educational resources. The findings reveal both benefits and challenges to mHealth app-EMR/EHR integration across different clinical settings. Most studies reported benefits of integration, including enhanced patient education and self-management, real-time data recording and sharing with clinicians, improved support for clinical decision-making, strengthened communication between patients and clinicians, and better patient health outcomes. However, several challenges were identified, including high drop-off rates in app usage, limited accessibility due to device restrictions, incompatibility between mHealth apps and EMR/EHR systems, increased clinical workload due to additional data integration, data accuracy concerns related to network connectivity, and data security issues.

### Implications

The study findings underscore the importance of addressing technical challenges and optimizing management processes to facilitate the successful integration of mHealth apps into EMR/EHR systems, ultimately leading to improved health care outcomes. Specifically, having patient health information such as blood glucose levels, blood pressure measurements, chemotherapy-related adverse events, body weights, and seizure records captured and automatically incorporated into EMR/EHR systems can significantly improve data accuracy and enhance the efficiency of provider workflows [13,17,22,23,27-30]. This also highlights that apps capture or log data specific to clinical needs, potentially requiring a level of customization. Easy access to these patient health data, along with system-generated summaries, reduces the time clinicians spend verbally discussing symptoms and signs with patients and caregivers [12,13,15,20,25,27,30]. This allows more time to be dedicated to discussing disease management strategies. Additionally, the ability to review health data before patient encounters enables clinicians to prioritize those most in need of attention, thereby improving both efficiency and the quality of patient care [13,28-30].

Patient involvement in the development and implementation of mHealth apps and its integration into EMR/EHR systems ensures that mHealth apps and the integration are tailored to their needs, preferences, and concerns, thereby enhancing usability, engagement, and trust [18,23,24,26,31]. The integration of mHealth app data into EMR/EHR systems provides significant benefits in several key areas [12,14,16,21,29]. It facilitates increased patient education by delivering timely and relevant information about health conditions and treatments. This educational feature empowers patients to take an active role in managing their health. mHealth apps also support self-management by allowing patients to track their health metrics, set goals, and receive personalized feedback. Enhanced communication with health care providers through these apps and EMR/EHR systems further improves patient engagement and adherence to treatment plans. As a result, patients are more likely to follow prescribed treatments, make necessary lifestyle changes, and achieve critical clinical outcomes, leading to an overall improvement in quality of life.

Additionally, the integration of mHealth app data with EMR/EHR systems has demonstrated notable benefits in improving patient access to health care services during the COVID-19 pandemic [13]. By seamlessly sharing patient data with health care providers, this integration facilitated remote consultations, continuous monitoring, and timely interventions, which were crucial in managing patient health during a period of widespread disruption [32].

### Literature Gap and Future Directions

The number of studies included in this review (n=19) is relatively small when compared with the vast array of mHealth apps currently in use. This suggests that research into the integration of mHealth apps with EMRs/EHRs and its impact on health care delivery and patient health outcomes is still in the early stages. Further comprehensive research is required to draw definitive conclusions in this area. Most studies examined third-party apps originally developed independently of the EMR/EHR ecosystem, with only a few focusing on native EMR/EHR-integrated apps (eg, Epic’s modules) [22,24]. The small number of studies on native EMR/EHR-integrated apps resulted in no substantial differences in impact being observed between these 2 types of apps in this review. This finding highlights the need for further research to explicitly investigate how third-party apps and native EMR/EHR modules interact with EMR/EHR systems differently and their respective effects on health care outcomes.

As for observational period, most studies have a short-term period, less than 1 year [12-16,18,20,21,23,25-27,29,30], indicating the need for longer study periods to confirm the persistence of effects. The cross-sectional design and small sample sizes of some studies limit the ability to establish cause-and-effect relationships between mHealth app usage and outcomes [14,16,17,21,30]. Additionally, most studies were conducted in hospital settings [12-15,20,21,23,26-29,31], while chronic diseases, such as diabetes and hypertension, are often managed in primary care settings [33]. Therefore, further studies are necessary to assess the integration and impact of mHealth apps in primary care settings. The focus on people with specific single chronic diseases [12-18,20-25,27-31] may limit the generalizability of research findings. Further research is needed to expand the scope to other health conditions, multimorbidity, or even disability, as these factors are often linked to poorer health outcomes and higher health care system costs [34,35].

Moreover, participants in most studies had good literacy [12-18,20-31] or were restricted to those proficient in the language [13,17,18,22-25,27,30]; this may reduce health equity for individuals with lower literacy levels or for people from linguistically diverse backgrounds. Given that people with lower literacy levels and those from culturally and linguistically diverse backgrounds often experience more disadvantage in accessing health care services [36,37] and are more likely to experience a poor outcome [38,39], health system reforms involving mHealth app integration with EMR/EHR systems should consider this and ensure that they have equitable access to health care. Efforts can be made to make mHealth services easily accessible to low-literacy individuals and to enhance individual health literacy through educational programs [40]. For people with low literacy, remote-monitoring devices that require little or no knowledge of mHealth app use from patients or carers may be a better option to capture their health data [41].

Finally, some studies excluded people who did not have a smartphone compatible with the app, who had moderate to severe cognitive impairment who owned a mobile phone, or who did not have internet access with their mobile phone [18,20,22,23,26,27], which may limit equitable access to mHealth apps. This exclusion could result in a digital divide, particularly affecting those without access to smartphones or other digital devices, such as older adults, people with lower socioeconomic status, or individuals in underserved communities. Expanding research to consider these populations is essential to ensure more inclusive health care reforms.

### Limitations

There are some limitations. First, this scoping review exclusively focused on academic literature published in English. The exclusion of non-English publications may result in potential bias, as relevant studies in other languages could have been overlooked. Second, most included studies were conducted in high-income countries. This may limit the generalizability of the findings to low- and middle-income countries, where health care systems, digital infrastructure, and patient populations may differ significantly.

### Conclusions

In summary, this scoping review provides the first comprehensive synthesis of published literature on the integration of mHealth app data into EMR/EHR systems and its impact on health care delivery and patient health outcomes. As the use of mHealth apps continues to rise, and people with diverse health conditions require increasingly personalized care, it is crucial that health care policy makers and administrators are equipped with evidence-based knowledge and guidance. Current literature remains limited, with included studies primarily focusing on populations with specific health conditions, short-term outcomes, and single hospital settings. Significant opportunities remain to build evidence based on the effectiveness of mHealth-EMR/EHR integration in improving long-term patient outcomes, such as rehospitalization, mortality, and cost, and assessing the scalability and sustainability of integration among more broader health conditions and disabilities across diverse health care settings.

## Supplementary material

10.2196/66650 Multimedia Appendix 1 Search string.

10.2196/66650 Multimedia Appendix 2 Study characteristics, mobile health apps, and their integration to electronic medical record or electronic health record systems.

10.2196/66650 Multimedia Appendix 3 Number of studies for each feature of mobile health apps and its integration into electronic medical record or electronic health record systems (note: detailed description of the features can be found in Multimedia Appendix 5.

10.2196/66650 Multimedia Appendix 4 Description of each feature of mobile health apps and their integration into electronic medical record or electronic health record systems.

10.2196/66650 Multimedia Appendix 5 Features of mobile health apps and their integration into electronic medical record or electronic health record systems.

10.2196/66650 Multimedia Appendix 6 Study findings, benefits, and challenges for the use of mobile health apps and their integration into electronic medical record or electronic health record systems on the health care delivery and patient health outcomes.

10.2196/66650 Checklist 1 PRISMA-ScR (Preferred Reporting Items for Systematic reviews and Meta-Analyses extension for Scoping Reviews) checklist.

## Abbreviations

EHR: electronic health record
EMR: electronic medical record
mHealth: mobile health
PRISMA-ScR: Preferred Reporting Items for Systematic reviews and Meta-Analyses extension for Scoping Reviews
