# Supplementary material for: Integrating Mobile Health App Data Into Electronic Medical or Health Record Systems and Its Impact on Health Care Delivery and Patient Health Outcomes: Scoping Review
Source: JMIR Mhealth Uhealth. 2025 Jun 23;13:e66650. doi: 10.2196/66650 (PMC12208509; doi:10.2196/66650)
Supplement: Multimedia Appendix 1 [file mhealth-v13-e66650-s001.docx]

# Search string (PubMed version).

| **Search** | **Query** |
| --- | --- |
| #1 | (“Mobile Applications” [MeSH term]) OR (mobile app* [Title/Abstract]) OR (mHealth app* [Title/Abstract]) OR (phone app* [Title/Abstract]) OR (digital app* [Title/Abstract]) OR (digital health app* [Title/Abstract]) OR (e-health app* [Title/Abstract]) OR (ehealth app* [Title/Abstract]) OR (health app* [Title/Abstract]) |
| #2 | (“Electronic Health Records” [MeSH term]) OR (electronic health record* [Title/Abstract]) OR (electronic medical record* [Title/Abstract]) OR (electronic health information [Title/Abstract]) OR (electronic medical information [Title/Abstract]) |
| #3 | #1 AND #2 |
| #4 | Restrict language to English |
| #5 | Restrict publication year to 2014-2024 |
