# Supplementary material for: Integrating Mobile Health App Data Into Electronic Medical or Health Record Systems and Its Impact on Health Care Delivery and Patient Health Outcomes: Scoping Review
Source: JMIR Mhealth Uhealth. 2025 Jun 23;13:e66650. doi: 10.2196/66650 (PMC12208509; doi:10.2196/66650)
Supplement: Multimedia Appendix 2 [file mhealth-v13-e66650-s002.docx]

# Study characteristics, mobile health applications (mHealth apps) and their integration to electronic medical/health record (EMR/EHR) systems.

| **First author, year, country** | **Study design** | **Study settings or data sources** | **Study population** | **App type** | **mHealth app and its integration into EMR systems** |
| --- | --- | --- | --- | --- | --- |
| Masiero 2024, Italy | Cohort | Division of medical senology and the division of pain therapy and palliative care of the European Institute of Oncology | 25 adult patients with diagnosed breast cancer and pain | Third-party | A PainRELife ecosystem consisted of a cloud technology platform interconnected with EHRs, which was named the Nu Platform, connected to the Fast Healthcare Interoperability Resources (FHIR) server for data analysis related to the patient care pathway. The Nu Platform was associated with a mobile app for patients named PainRELife, which collected health care data. This technological solution permitted dual communication between patients and health care professionals. Information collected by the mobile app was saved in the Nu Platform and overseen by health care professionals. |
| Solomon 2024, US | Cross-sectional | One large US-based academic medical center and its affiliated rheumatology practices | 300 adult patients with rheumatoid arthritis (App user group: 150; Control group: 150) | Third-party | A mobile app that was integrated into the EHR, allowing rheumatologists to easily view the patient-reported outcomes (PROs) data. Four validated PROs short forms were delivered to patients through the application with SMS push notification reminders, one PRO every 48 hours; thus, all four PROs were requested in an 8-day cycle. The four PROs included were PRO Measurement Information System (PROMIS) Pain Interference, PROMIS Fatigue, PROMIS Function, and the RA Disease Activity Index. |
| Huang 2023, China | Cross-sectional | Clinical Data Analysis and Reporting System of the Hong Kong Hospital Authority, which contains health information of all public hospitals and clinical settings | 109,823 adult patients with type 2 diabetes mellitus who had received annual assessment for diabetes complications (non-users of the eHealth app: 76,356; eHealth app: 31,723; eHealth app with the management module: 1,744) | Third-party | A mobile app leveraged the advantages of the electronic health Record Sharing System (EHRSS). The EHRSS aimed to provide a free and lifelong EHRs for the general population by enabling a two-way sharing system among public and private clinicians for healthcare purposes. Major functions of the app included the provision of general public health information and news, and the viewing of the users’ health records (medications, allergies, vaccine records, etc.). The subsequent introduction of the Health Management Module allowed patients to record their blood pressure, blood sugar, and heart rate in the app to monitor the change in their health conditions. A reminder function was set up to alert users in regular measurement of their health indices. |
| Young 2023, US | Mixed method | A community general neurology practice at the Mayo Clinic, Rochester, Minnesota | 171 adult patients with migraine (App user group: 121; Control group: 62) | Native EMR/EHR functionality | An EHR- and mobile app-integrated Migraine Interactive Care Plan (MICP) that was designed using the existing EHR functionality (Epic Systems Corporation; Epic MyChart Care Companion module) and delivered through a mobile app on the patient’s smartphone or tablet. Patients submitted symptom assessments and physiologic data through the app, and the results were available in the EHR and through care team dashboards. |
| Crossen 2022, US | Cohort | University of California, Davis medical center | 40 paediatric patients with type 1 diabetes (Intervention group: 20; Control group: 20) | Third-party | Patient glucose data were transmitted via Bluetooth from the meter to the mobile device’s OneTouch Reveal app, then via Apple Health to Tidepool (a secure web-based platform for diabetes data management) and Epic MyChart apps (the EMR used at the medical center) on the same device. When the device connected to Wi-Fi, these apps transmitted data to the cloud, making them viewable by the providers in Epic (via a flowsheet within the patient’s chart) and Tidepool (via the patient profile, which was linked to the clinic’s account). |
| Lee 2022, South Korea | Three-arm RCT | Two separate university-affiliated hospitals | 269 adult patients with type 2 diabetes (Usual care: 87; Mobile diabetes self-care [MC]: 91; MC with personalized, bidirectional feedback from physicians: 91) | Third-party | A mobile app that allowed patients to enter their self-care data (SMBG, dietary habits, and step count), and automated text messages (educational, behavioral, and motivational messages) from the iCareD system were sent to their mobile phones. The app was integrated with the EMR in each hospital; therefore, health care providers also evaluated participants at every 3-month visit based on the data obtained from the mobile app. |
| Morgenthaler 2022, US | Cohort | Center for Sleep Medicine | 222 adult patients with chronic insomnia disorder | Third-party | A cognitive behavioral therapy for insomnia program that was delivered via personal mobile phone devices and integrated with EHR. It contained symptom assessments (questionnaires), a daily sleep diary (flowsheet), reminders, and targeted education, with the goal of empowering patients to self-manage their condition. |
| Stan 2022, US | Case series | Breast oncology practice of a large academic medical center | 23 adult breast cancer survivors | Native EMR/EHR functionality | An interactive care plan (referred to as Epic’s MyChart® Care Companion) was a EHR-integrated tool, embedded within the Mayo Clinic mobile application, which can be accessed through a patient’s mobile device via their patient online services (portal) account. |
| Agnihothri 2021, US | Cohort | A hypertension clinic | 1633 adult patients attending the hypertension clinic (App user group: 726, Control group: 907) | Third-party | A EMR-integrated app enabled patients to upload readings via BP measurement devices, and continuously communicate data on their vitals while the provider monitored, intervened and gave timely feedback. The app allowed patients to request refills and medication changes, and sent a summary document automatically to the patient’s EMR so that patients can have a macro view of their readings. |
| Choi 2021, South Korea | Cohort | Seoul National University Bundang Hospital | 99 paediatric patients with epilepsy or caregivers of children (App user group: 51; Rare/Non-user group: 48) | Third-party | A mobile epilepsy management app, called Brain4U, which was integrated into hospital EHRs. App data was mapped to Fast Healthcare Interoperability Resources (FHIR) elements. The app with functions including a seizure diary, medication reminders that can provide adherence data, comorbidity self-screening tools, and a personal dashboard for each individual patient, as well as educational material. |
| Lewis 2020, UK | Two-arm RCT | Mental Health Trusts in Manchester and South London | 81 adults with severe mental illness (schizophrenia and related disorders) (App enhanced monitoring plus standard care: 40, Standard care: 41) | Third-party | A smartphone-based platform (ClinTouch) aimed to help persons with severe mental illness to manage their symptoms and prevent relapse. It integrated into NHS Trust information and communication technology platforms, which enabled the streaming of summary information into EHRs and enabled health professionals to track current symptoms on desktops at the team base and receive personalized alerts when symptoms exceeded a pre-agreed threshold. |
| Guo 2019, China | Quasi-experimental | Subei People’s Hospital | 66 adults with a history of chronic heart failure (CHF) of ≥3 months | Third-party | A mobile app, created on both Android and iOS platforms, was developed for reporting health information, monitoring clinical signs, and enabling direct supervision and instruction for patients. Participants can use the app at home to record and upload data related to the risk of CHF self-care management, including daily recording of symptom and sign changes and medication adherence. It also linked to weight scale, a blood pressure (BP) monitor with a cuff, multicomponent remote monitor (for an electrocardiogram [ECG], peripheral capillary oxygen saturation, and BP), long-term wearable ECG monitor (BECG1200-A, Thoth Medical Technology Co, Ltd, Suzhou, China), and mobile phone ECG monitor (Zhongwei Laikang Technology Development Co, Ltd, Beijing, China). The integrated remote monitoring service platform is a cloud-based, tablet computer–accessed, secure Web platform to collect and integrate data. The EMR of each participant was recorded on the remote monitoring service platform. |
| Kim 2019, South Korea | Three-arm RCT | Outpatient clinic of Seoul National University Bundang Hospital | 43 adult patients with obstructive sleep apnoea (App and wearable device group: 15; App only group: 15; Control group: 13) | Third-party | A mobile app, MyHealthKeeper (Samsung Electronics Co, Seoul, South Korea), was compatible with health data collection platforms of private companies and linked to hospital EMR system, Bestcare (ezCaretech Co, Seoul, South Korea). The app allowed patients recorded their sleep data via the Samsung Charm wearable activity tracker band. |
| Weatherly 2019, US | Mixed method | Stanford Children’s Health diabetes clinics | 32 paediatric patients with type 1 diabetes, or their parents | Third-party | An Accu-Chek Connect App, paired the glucose meters with the app, set up the Apple Health App (Apple, Cupertino, California) in a Apple iPod or iPhone (5s or higher), and linked the Accu-Chek Connect App with the Apple Health App through HealthKit. The glucose meter data were transmitted directly to the Epic EHR through Wi-Fi. |
| Bae 2018, South Korea | Two-arm RCT | Six institutions within the Korean SouthWest Oncology Group | 101 adult patients who received ≥2 cycles of chemotherapy, and were scheduled for two more cycles | Third-party | An electronic patient-reported outcome smartphone (PRO-SMART)-related app allowed patients to enter their PRO measures, including symptomatic adverse events associated with chemotherapy. An analysis report of cumulative PHR data is provided to the clinician in a format suitable for upload to EMRs. |
| Cho 2018, South Korea | Two-arm RCT | A sleep clinic of a tertiary center | 59 adult patients with habitual snoring or witnessed apnoea (App user group: 24; Control group: 23) | Third-party | A smartphone app that was linked to a hospital EMR system to encourage an interactive lifestyle modification approach between physicians and patients. The app was composed of two modules: a diet and a physical activity module. The diet module recorded daily dietary intake. The physical activity module recorded the number of steps through a wrist-worn activity tracker (The Misfit Shine; Misfit, Burlingame, CA, USA). |
| Ryu 2017, South Korea | Two-arm RCT | Seoul National University Bundang Hospital | 68 adult patients with a body mass index of over 23 kg/m^2^ (App user group: 44; Control group: 24) | Third-party | An Android operating system-based mobile phone app (MyHealthKeeper) designed to collect health-related lifestyle data via the wearable activity device (Misfit Shine; Misfit Wearables Corporation, Burlingame, CA, USA). The MyHealthKeeper interface design showed patient-generated lifestyle data in a graphical format on a clinician’s EHR screen. |
| Kumar 2016, US | Qualitative study | A paediatric diabetes clinic | 10 paediatric patients with diabetes or parents of paediatric patients | Third-party | An iOS Epic MyChart mobile app was integrated into the Epic EHR system (Verona, WI, USA). It collected patients’ blood glucose data from the continuous glucose monitor device (Dexcom, San Diego, CA, USA) via Health app. Data was visualised and assessed by clinicians and providers can sent messages from EHR to patients/parents’ app. |
| Kim 2014, South Korea | Cohort | Seoul St. Mary’s Hospital | 70 adult outpatients with type 2 diabetes mellitus (App user group: 35; Control group: 35) | Third-party | A mobile app (Galaxy S-II Smartphone, Samsung Electronics, Suwon, Korea) allowed patients to check and record their blood glucose data using CareSens-LINK blood glucose monitor (i-SENS, Wonju, Korea). The data were then automatically transferred to the hospital due to app integrated to EMR. The medical staff analyzed the data and sent recommendations and feedback tailored to the patient an average of once per week. |
