# Supplementary figures and images for: Integrating Mobile Health App Data Into Electronic Medical or Health Record Systems and Its Impact on Health Care Delivery and Patient Health Outcomes: Scoping Review
Source: JMIR Mhealth Uhealth. 2025 Jun 23;13:e66650. doi: 10.2196/66650 (PMC12208509; doi:10.2196/66650)

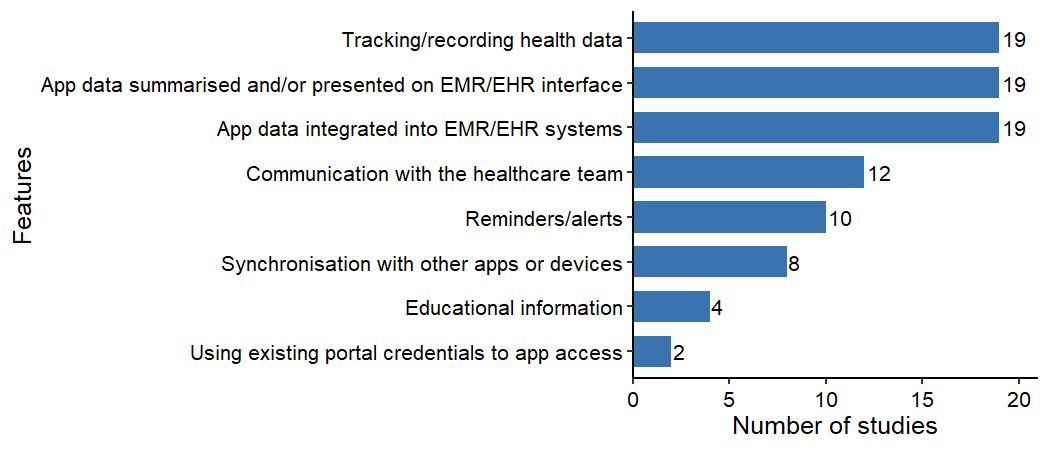

Supplement: Multimedia Appendix 3 [file mhealth-v13-e66650-s003.png]
