# Supplementary material for: Integrating Mobile Health App Data Into Electronic Medical or Health Record Systems and Its Impact on Health Care Delivery and Patient Health Outcomes: Scoping Review
Source: JMIR Mhealth Uhealth. 2025 Jun 23;13:e66650. doi: 10.2196/66650 (PMC12208509; doi:10.2196/66650)
Supplement: Multimedia Appendix 4 [file mhealth-v13-e66650-s004.docx]

# Description of each feature of mobile health applications (mHealth apps) and their integration into electronic medical/health record (EMR/EHR) systems.

| **Feature** | **Description** |
| --- | --- |
| Tracking/recording health data | Tracks or records user’s measurements, such as blood glucose, blood pressure, weight, pulse, temperature,  mood, and sleep patterns, symptoms of disease, and side effects. |
| Synchronisation with other apps or devices | The app has the ability to synchronise itself and the entered data with another mobile  app or device (e.g. Apple Watch). |
| Educational information | The app provides users with health-related information, such as details about health conditions, management information, treatment guidelines, preventive care information, symptom tracking guidance, and health literacy resources. |
| Reminders/alerts | The app has the ability to remind users to take medications or enter data, or to alert users that their measurements fall outside the normal range. |
| App data integrated into EMR/EHR systems | Health-related data collected or stored in a mobile health app is seamlessly transferred or shared with a provider’s electronic medical/health system. |
| Access app with existing portal credentials | Patients don’t need to manage multiple logins. They can use their existing portal credentials to access the app, simplifying the authentication process. |
| Communication with the healthcare team | The app allows users to communicate with their healthcare team through text messages, chat boxes, voicemails, or phone calls. |
| App data summarised and/or presented on EMR/EHR interface | The patient's health data is summarised and/or displayed as text or graphs for users and/or healthcare providers. |
