# Supplementary material for: Integrating Mobile Health App Data Into Electronic Medical or Health Record Systems and Its Impact on Health Care Delivery and Patient Health Outcomes: Scoping Review
Source: JMIR Mhealth Uhealth. 2025 Jun 23;13:e66650. doi: 10.2196/66650 (PMC12208509; doi:10.2196/66650)
Supplement: Multimedia Appendix 5 [file mhealth-v13-e66650-s005.docx]

# Features of mobile health applications (mHealth apps) and their integration into electronic medical/health record (EMR/EHR) systems.

| **First author, year, country** | **Tracking/recording health data** | **Synchronisation with other apps or devices** | **Educational**  **information** | **Reminders/alerts** | **App data integrated into EMR/EHR systems** | **Using existing portal credentials to app access** | **Communication with the healthcare team** | **App data summarised and/or presented on EMR/EHR interface** |
| --- | --- | --- | --- | --- | --- | --- | --- | --- |
| Masiero 2024, Italy | √ |  | √ |  | √ | √ | √ | √ |
| Solomon 2024, US | √ |  |  | √ | √ |  | √ | √ |
| Huang 2023, China | √ |  |  | √ | √ |  |  | √ |
| Young 2023, US* | √ |  | √ |  | √ |  |  | √ |
| Crossen 2022, US | √ | √ |  |  | √ |  | √ | √ |
| Lee 2022, South Korea | √ | √ |  | √ | √ |  | √ | √ |
| Morgenthaler 2022, US | √ |  | √ | √ | √ |  |  | √ |
| Stan 2022, US* | √ |  | √ | √ | √ |  | √ | √ |
| Agnihothri 2021, US | √ |  |  | √ | √ |  | √ | √ |
| Choi 2021, South Korea | √ |  |  | √ | √ | √ |  | √ |
| Lewis 2020, UK | √ |  |  | √ | √ |  | √ | √ |
| Guo 2019, China | √ | √ |  | √ | √ |  | √ | √ |
| Kim 2019, South Korea | √ | √ |  |  | √ |  |  | √ |
| Weatherly 2019, US | √ | √ |  | √ | √ |  | √ | √ |
| Bae 2018, South Korea | √ |  |  |  | √ |  |  | √ |
| Cho 2018, South Korea | √ | √ |  |  | √ |  |  | √ |
| Ryu 2017, South Korea | √ | √ |  |  | √ |  | √ | √ |
| Kumar 2016, US | √ | √ |  |  | √ |  | √ | √ |
| Kim 2014, South Korea | √ |  |  |  | √ |  | √ | √ |

* Native EMR/EHR-integrated apps.
