# Supplementary material for: Integrating Mobile Health App Data Into Electronic Medical or Health Record Systems and Its Impact on Health Care Delivery and Patient Health Outcomes: Scoping Review
Source: JMIR Mhealth Uhealth. 2025 Jun 23;13:e66650. doi: 10.2196/66650 (PMC12208509; doi:10.2196/66650)
Supplement: Multimedia Appendix 6 [file mhealth-v13-e66650-s006.docx]

# Study findings, benefits, and challenges for the use of mobile health applications (mHealth apps) and their integration into electronic medical/health record (EMR/EHR) systems on the healthcare delivery and patient health outcomes.

| **First author, year, country** | **Main study findings** | **Benefits/strengths** | **Challenges/limitations** |
| --- | --- | --- | --- |
| Masiero 2024, Italy | Patients with breast cancer who used the mobile app reported reduced pain intensity at 3 months (P=0.04). The total number of times the app was accessed was positively correlated with pain intensity at 3 months (P=0.03). | (1) The mobile app enables patient education and the collection of patient-reported outcomes. (2) The app permits the achievement of critical clinical outcomes and improvement in quality of life. (3) The health-integrated ecosystems helped reduce the burden on health care professionals and optimizing health system resources. | (1) Longer study period is needed to ensure the effect remains. |
| Solomon 2024, US | No differences were found in the volume of participant visits to rheumatologists for rheumatoid arthritis or visit delays between app user group and the control group. | (1) It is feasible to implement an EHR-integrated patient-reported outcomes mobile app that was used by patients and clinicians. | (1) This new technology is difficult for clinicians to integrate into their clinical workflow and use for clinical and scheduling decisions. (2) Although education was provided to clinicians and their staff on how to use the new data and the EHR in-basket messages, this type of practice redesign may take more intense education over a longer period. |
| Huang 2023, China | Users of the eHealth Management Module were found to have more optimal glycated haemoglobin (HbA1c) levels across all subgroups, with the strongest effect observed in younger females (adjusted Odds Ratio [aOR]=1.66, 95% Confidence Interval [CI]: 1.27-2.17). eHealth App usage is also positively associated with optimal HbA1c levels, particularly amongst younger females (aOR=1.17, 95% CI: 1.08-1.26). | (1) Use of eHealth mobile app specifically targeting the management of diabetes through behavioural interventions and recommended lifestyle changes have been proven beneficial across the population. (2) These findings support its potential adoption in diabetes patients. | (1) The cross-sectional nature of the study makes it impossible to establish the cause-and-effect relationship between eHealth usage and glycaemic control. (2) Future studies should examine the impact of eHealth interventions on other clinical targets and diabetes complications. |
| Young 2023, US* | The percentage of patients reporting favourable satisfaction increased from a baseline of 35% to 83% over the first 6 months. Fewer face-to-face visits were observed in the EMR- and mobile app–integrated Migraine Interactive Care Plan (MICP) group (10.7%) compared with controls (42%; P<0.001). | (1) The implementation of an EHR-integrated mobile app-based care plan for migraine in a community neurology practice was successful. (2) The MICP has the potential to improve patient access without increasing care team workload and the need for patient input from diverse populations to improve and sustain patient engagement. | (1) Additional studies are needed to assess its impact in primary care. |
| Crossen 2022, US | Families in the intervention group were more likely than those in the control group to review their glucose data using mHealth apps after 1 month (P<0.001), but by 6 months, this difference had disappeared. Perceived ease of contacting the clinical team for assistance was lower for the intervention group after 6 months (when receiving usual care) in comparison to during the intervention period (P=0.48) and compared with a control group who did not have exposure to remote monitoring (P=0.03). | (1) The use of remote monitoring accelerated patients’ and caregivers’ adoption of mHealth apps for the self-review of glucose data during the first month after diagnosis. (2) Remote glucose monitoring was found to improve the efficiency of provider workflows. (3) This study provided a proof-of-concept for importing glucose data directly into the EHR, but also demonstrated that the data relay was simpler to establish, and the visualization of data was superior via a diabetes-specific data platform, compared with the EHR. | (1) Difficulties establishing remote monitoring via Epic resulted from problems creating full access MyChart accounts for 3 participants due to medical-center-specific policies, rather than technical errors. (2) The patient must have a personal mobile device with Bluetooth and internet capability, which can download and run multiple digital health apps simultaneously. (3) The data relay into the EHR in this study required the use of Apple Health and therefore could only be performed using an Apple device. |
| Lee 2022, South Korea | At 12 weeks after the intervention, the mean decline in HbA1c levels was significantly different among the 3 groups (Usual care vs Mobile diabetes self-care [MC] vs MC with personalized, bidirectional feedback from: −0.49% vs −0.86% vs −1.04%; P=0.02). The HbA1c level decreased in all groups; however, it did not differ among groups after 26 weeks. | (1) mHealth interventions support self-care and diabetes education and encourage lifestyle modification. (2) Mobile apps that receive blood glucose data from a connected glucometer are available and have the capacity to make data upload and review less burdensome. (3) Automated integration of glucose and lifelog data in the EMR between scheduled clinic visits improves the provider workflow for reviewing data and improves communication with patients, leading to better care. | (1) Age is a barrier to digital health care adoption and may influence the adoption of new technologies. |
| Morgenthaler 2022, US | Sleep efficiency improved from baseline until the completion of the program (P=0.001), and the Insomnia Severity Index improved (P=0.006). | (1) There were significant improvements in both the Insomnia Severity Index scores and sleep efficiency among patients who remained engaged with the care plan. (2) The integration of the patient-facing app with the provider accessible EHR is a unique feature, and the study did not indicate a substantial increase in administrative burden. | (1) The app has an average dropout rate of 24.7%. |
| Stan 2022, US* | The monthly average task completion rates were 62% for the quality of life (QOL) questionnaire, 59% for symptom assessments, and 37% for activity reminders. Task completion rate decreased over time. Eleven of 253 symptoms and QOL questionnaires (4.3%) generated messages for care escalation. | (1) The care plan may be ready for larger scale implementation in the oncology practice. | (1) Gradual decrease in response rate over time, participation drops off quickly after enrolment in studies evaluating digital health solutions, which may be due to the time commitment involved, suboptimal user interfaces, loss of the novelty factor over time, and perceived lack of the effectiveness of the intervention for the needs of the participants. |
| Agnihothri 2021, US | Heavier app use is associated with a reduced blood pressure (BP). The drop was higher for sicker patients. Patients who adopted the app, compared with those who did not, reduced their systolic BP by 1.89mmHg and diastolic BP by 0.87mmHg on average and stage 2 hypertension patients by 4.01mmHg systolic and 4.37mmHg diastolic on average. | (1) Using mHealth in a private clinical practice can improve patient-provider coordination, enable customised care and could be instrumental in managing patient BP. (2) Higher upload frequency was associated with greater reduction in BP. If a patient uploads their readings periodically into the app, that patient enables more frequent monitoring and enables more opportunities to receive communication from the physician and his support staff. (3) mHealth technology therefore helps in reducing information asymmetry, improves coordination between the patient and the providers, and improves patient engagement. | (1) There was uncertainty around operational implications of using such an app in practice. As the interventions (97% during a 3-month study period) can be handled by dietitians and medical assistants, the responsibility of patient monitoring shifts from the physician to the support staff. |
| Choi 2021, South Korea | Adherence rates from mobile app were highly varied (mean 34.8 ± 39.7%, range 0-100%). Half of study participants had adherence rates over 70%. The app-user group demonstrated significant improvement in their self-knowledge of epilepsy, including their seizure triggering factors (p=0.004) and adverse events related to anti-seizure medication (ASM) (p=0.039), compared to the baseline assessments. There were no significant improvements in the rare/nonuser group. About 70% of the participants reported that the app was helpful in managing their own or their children’s epilepsies. Caregivers mentioned educational content and medication reminder as the most helpful features. | (1) Mobile health technology can assist patients and caregivers to record their healthcare data and aid in self-management. It can help obtain precise seizure records, seizure triggering factors, and adverse reactions of ASM from apps. (2) Mobile health technology linked to EHRs offers opportunities for sharing data efficiently with the patient’s clinicians. (3) In this study, reminders of when to take medication was considered a useful feature among the app users and may assist treating clinicians in monitoring their patient’s compliance with medication. | (1) The drop-off rate in app usage was high by patients. (2) The accuracy of using mobile app to measure adherence to medication depends on patient app using behaviours. |
| Lewis 2020, UK | At 12 weeks, 90% of those in the active monitoring group continued to use the system and exhibited an adherence rate of 84%. Active symptom monitoring was associated with no difference on the empowerment scale in comparison to the usual management group at 12 weeks. The intent-to-treat analysis showed a significant reduction in the active symptom monitoring group over 12 weeks in the early intervention center. Alerts for personalized early warning signs of relapse were built into the workflows of both National Health Service (NHS) Trusts. Qualitative analyses supported the acceptability of the system to participants and staff. | (1) The mobile app was safe and acceptable. (2) It provided opportunities for patients to record symptoms every day, which can reduce the confounding effects of retrospective recall bias, forgetting, and averaging in symptom appraisal. (3) It allows the context of symptom changes to be assessed and increases patient involvement in continuing care through participation in symptom and progress monitoring. (4) With symptom data streamed into the EHR system, health professionals could view it on a secure desktop at the team base. (5) Alerts for early warning signs were built into the workflows of the two NHS Trusts. | (1) The commercial provider of the EHR in the second Trust, did not comply with the study, indicating a potential barrier to full scale roll out in the NHS where Trusts have a range of different commercially provided EHR platforms. (2) At the time of the trial (2014-2016), the app was only available for the Android operating system. (3) Artifacts in functionality were identified for improvement, such as alerts being mistimed if the user was temporarily in an area without a wireless network. |
| Guo 2019, China | Patients with chronic heart failure (CHF) indicated that they were generally satisfied with the intervention for its ease of use and usefulness. More than 91% of physicians believed the program was effective, and 87% of physicians stated that their professional knowledge could always be refreshed and enhanced through a library hosted on the platform and remote consultation. More than 60% of participants showed good adherence to the care plan in the study period, and 79% of patients maintained a consistent pattern of reporting and viewing their data over the course of the 4-month follow-up period. The program showed a positive effect on self-management for patients (healthy diet: P=0.046, more fruit and vegetable intake: P=0.02, weight monitoring: P=0.002, blood pressure: P<0.001, correct time: P=0.049, and daily dosages of medicine taken: P=0.006). | (1) The CHF management team was key in engaging patients to participate the program; the benefits of the program also include support in recording and tracking health status, encouragement and reassurance received from medical staff, timely detection, recognition and management of subtle changes in the condition, and more convenient and faster communication among all participants. (2) Some physicians perceived the platform to be effective as a communication tool to share data in a timely, accurate, and visual manner, so that they can be armed with all relevant health information contained in one system, especially in an emergency or unfamiliar health care setting, for care planning. | (1) Because of technical restriction, semiautomatic input of data in this program greatly wastes human resources and possibly increases the error rate. |
| Kim 2019, South Korea | The body weight showed a significant reduction in the 2 intervention groups after intervention, and the mobile app-only group showed more weight loss compared with the controls (P=0.01). There were no significant changes in sleep-related health outcomes. | (1) The study revealed that the reduction of weight was because of the use of apps, not the use of the wearable devices. (2) With EHR-tethered system, clinicians are available to show their patient’s daily lifestyle and to deliver coaching feedback. | (1) This study could not provide a longitudinal observation of the  EHR-tethered app system because of the practical constraints. (2) Due to the short clinical trial period and the small number of study participants, it was difficult to determine a causal  Relationship. (3) The study did not provide information about  the precise improvement in the health outcomes of app users. |
| Weatherly 2019, US | The number of patient-initiated calls (P=0.23) and HgbA1c values (P=0.08) did not improve, nor was there a clinically significant change in the number of blood glucose checks per day. | (1) Patients demonstrated an interest in further development of such technology. | (1) Although integration is technically possible, the patient experience was cumbersome and resulted in low adherence to the technology. (2) One participant expressed burnout with diabetes care, rarely checked blood glucose, and did not want this ‘‘tracked’’ in the EHR. (3) There were fewer participant-initiated contacts during the learned phase, which may have been because participants had a better sense of how to manage their blood glucose. |
| Bae 2018, South Korea | The number of symptomatic adverse events (AEs) related to chemotherapy recorded in EMRs (mean ± standard deviation [SD]) increased from 0.92 ± 0.80 to 2.26 ± 1.80 (P<0.001), and grading of AEs increased from 0.81 ± 0.69 to 1.00 ± 0.62 (P=0.029). After using the app, numeric rating scale for pain (mean ± SD) increased from 0.20 ± 0.72 0.99 ± 1.55 (P<0.001). A patient-reported questionnaire revealed that 64.2% of patients found it useful and 83% found it easy to use. | (1) The mobile app is feasible and accessible for assessment of symptomatic AEs in cancer patients receiving chemotherapy. (2) It provides patient-reported outcome (PRO) data as a summary presented as text and graphs that a clinician can review a glance, allowing appropriate identification of symptoms within a short period of time. (3) The use of innovative technological systems provides an affordable solution to the increasing demands placed on self-care activities. (4) The reason for the high rate of accessibility is that the app is an icon- and image-based system that is easy to use at any time and any place. | (1) The app did not lead to improved symptom management or improved patient satisfaction with their overall management. This is because the medical staff did not perform remote monitoring of personal health record data entered by the patient and did not implement timely interventions based on these data. (2) The drop-off rate could not be analysed because patients enter their personal health record voluntarily and irregularly. |
| Cho 2018, South Korea | The success rate of weight loss was significantly higher in the app user group when compared to the control group (22/24 vs. 9/23; P<0.001). Although obstructive sleep apnoea parameters showed a tendency for a decrease in both groups, apnoea hypopnea index, respiratory distress index, and oxygen desaturation index had not been improved significantly. | (1) The linkage between the hospital EMR and the mobile app enables all information on the patient’s diet and physical activity summarised on the physician’s EMR interface. This allows physicians to check the parameters and show a summary of the lifestyle data to their patients, encouraging further lifestyle modifications based on these data. (2) The linkage also facilitates refining and adjusting personalized activity and dietary goals until the following session. | (1) Longer study period is needed to ensure the effect remains. |
| Ryu 2017, South Korea | The mobile app group showed significantly higher weight loss than the control group (mean 1.4 kg, 95% CI: 0.9-1.9; P<0.001) at week 4. In addition, triglyceride levels were significantly lower by the end of the study period (mean 2.59 mmol/L, 95% CI: 17.6-75.8; P=0.002). | (1) App use correlated significantly with larger changes in body weight and clinical parameters, signifying a better health status than with conventional treatment. (2) This study showed that patients actively used the app to improve the doctor-patient relationship. (3) The innovative EHR-tethered app-based system that allowed clinicians and patients to share lifelog data. | (1) This study could not provide a longitudinal observation of the EHR-tethered app system. (2)  The study did not provide information about the precise improvement in the health outcomes of app users. |
| Kumar 2016, US | The pediatric endocrinologist reported that after successful enrollment, the system enabled secure communication, timely access to information, and enhanced  interpretation of large volumes of patient device data. | (1) Passive EHR-based data delivery, coupled with automated triage and intuitive visualization, facilitates  more efficient provider workflow for reviewing data and improved. (2) The app not only served as the infrastructure for sharing data, but simultaneously facilitated secure discussion among adolescents, parents, and providers  communication with patients. | (1) Implementation on a larger scale  of providers and patients is necessary to demonstrate impact on diabetes outcome measures. (2) The app was only available with Apple devices. |
| Kim 2014, South Korea | Both mobile app user group and control group showed a tendency towards a decrease in the mean HbA1c level after 3 months (7.7% to 7.5%, P=0.077). In the more app satisfied group, the mean HbA1c level decreased from 7.7% to 7.3% (P=0.001), whereas in the less satisfied group, the mean HbA1c result increased from 7.7% to 8.1% (P=0.062), showing mean values much worse than that of the no-smartphone control group (from 7.7% to 7.7%, P=0.093). | (1) It is essential to provide the patient with a well-functioning high-quality tool capable of increasing patient satisfaction and willingness to use. | (1) Commercialise a medical feedback-enabled mobile phone involves human-resource costs for the medical team that provides the medical feedback. (2) An app is difficult to apply or when encountering frequent device breakdowns that affect compliance reduced user satisfaction. (3) Patient satisfaction plays a crucial role in blood glucose management. |

* Native EMR/EHR-integrated apps.
